# Circulating TIMP-1 is associated with hematoma volume in patients with spontaneous intracranial hemorrhage

**DOI:** 10.1038/s41598-020-67250-9

**Published:** 2020-06-25

**Authors:** Manuel Navarro-Oviedo, Roberto Muñoz-Arrondo, Beatriz Zandio, Juan Marta-Enguita, Anna Bonaterra-Pastra, Jose Antonio Rodríguez, Carmen Roncal, Jose A. Páramo, Estefania Toledo, Joan Montaner, Mar Hernández-Guillamon, Josune Orbe

**Affiliations:** 1 Laboratory of Atherothrombosis, CIMA, Universidad de Navarra, Instituto de Investigación Sanitaria de Navarra, IdisNA, Pamplona, Spain; 2 Neurology Service, Complejo Hospitalario de Navarra, IdisNA, Pamplona, Spain; 3 Neurovascular Research Laboratory, Vall d´Hebron Research Institute, Universitat Autònoma de Barcelona, Barcelona, Spain; 4 Centro de Investigación Biomédica en Red en Enfermedades Cardiovasculares (CIBERCV), Instituto de Salud Carlos III, Madrid, Spain; 5 Haematology Service, Clínica Universidad de Navarra, Pamplona, Spain; 6 Department of Preventive Medicine and Public Health, School of Medicine, Universidad de Navarra, IdiSNA, Pamplona, Spain; 7 Centro de Investigación Biomédica en Red en Fisiopatología de la Obesidad y Nutrición (CIBEROBN), ISCIII Madrid, Spain

**Keywords:** Molecular biology, Neuroscience, Biomarkers, Neurology

## Abstract

Matrix metalloproteinases (MMPs) are proteolytic zinc-endopeptidases regulated by tissue Inhibitors of matrix metalloproteinases (TIMPs). We evaluated the potential of MMPs and TIMPs as clinical tools for Intracranial Haemorrhage (ICH). Spontaneous non‐traumatic ICH patients were recruited from two hospitals: Complejo Hospitalario de Navarra (CHN = 29) and Vall d´Hebron (VdH = 76). Plasmatic levels of MMP-1, −2, −7, −9, −10 and TIMP-1 and their relationship with clinical, radiological and functional variables were evaluated. We further studied the effect of TIMP-1 (0.05–0.2 mg/Kg) in an experimental tail-bleeding model. In CHN, TIMP-1 was associated with admission-hematoma volume and MMP-7 was elevated in patients with deep when compared to lobar hematoma. In VdH, admission-hematoma volume was associated with TIMP-1 and MMP-7. When data from both hospitals were combined, we observed that an increase in 1 ng/ml in TIMP-1 was associated with an increase of 0.14 ml in haemorrhage (combined β = 0.14, 95% CI = 0.08–0.21). Likewise, mice receiving TIMP-1 (0.2 mg/Kg) showed a shorter bleeding time (p < 0.01). Therefore, the association of TIMP-1 with hematoma volume in two independent ICH cohorts suggests its potential as ICH biomarker. Moreover, increased TIMP-1 might not be sufficient to counterbalance MMPs upregulation indicating that TIMP-1 administration might be a beneficial strategy for ICH.

## Introduction

Stroke is the second leading cause of death and disability worldwide^1^. Intracranial hemorrhage (ICH), accounting for 10–15% of all strokes, is the most devastating subtype^2^. However, unlike ischemic stroke, the incidence of ICH has not declined over the last decades^3^ and no specific treatments have been developed^4^. Almost 40% of ICH patients will die within the first 30 days and only 20% of survivors will achieve independent functionality^5^.

ICH is a complex disease characterized by blood vessel rupture and extravasation into brain parenchyma, leading to hematoma expansion, ischemia and neuronal loss^6^. The most common ICH are either lobar or deep in location and differ in admission hematoma volume, hematoma growth and outcome^7^. Several factors are involved in the ICH pathophysiology including: (a) the remodeling of the extracellular matrix (ECM) which in physiological conditions maintains the integrity, elasticity and strength of the arterial wall, (b) the breakdown of the blood-brain barrier (BBB) which contributes to edema formation and neuronal degeneration and (c) the inflammatory responses involved in secondary brain injury^8,9^.

Matrix metalloproteinases (MMPs) are zinc-dependent endopeptidases with proteolytic activity that contribute to ECM degradation, tissue remodeling and regeneration^10^. The catalytic activity of MMPs is regulated by tissue inhibitors of matrix metalloproteinases (TIMPs)^11^. The four members of the TIMP family (TIMP 1–4) are broad spectrum inhibitors that directly bind to the catalytic domain of MMPs^12^.

After brain injury, the role of MMPs and their inhibitors may be critical, since both participate in early acute injury along with tissue recovering during later stages^13^. Clinical studies have observed increased MMP-2, −3, −9 and TIMP-1 levels in blood and cerebrospinal fluid of ICH patients when compared to normal population^14,15^, suggesting that MMPs/TIMPs are likely to be involved in brain injury after spontaneous ICH and may be associated with patients’ outcome^16^. In the last years our group has studied the role of MMPs in different neurovascular diseases. We showed that TIMP-1 levels were associated with mortality in patients with traumatic brain injury and malignant middle cerebral artery occlusion^17–19^, while MMP-2 expression might be implicated in the development of amyloid angiopathy in ICH patients^20^. Moreover, MMPs have been related to hematoma expansion, edema growth and neuronal loss in extensive pre-clinical studies^16^. However, their potential as therapeutic or diagnostic clinical tools has not yet been fully identified.

In this study, we explored the plasmatic levels of several MMPs and TIMP-1 and their relationship with hematoma volume and location as well as with other clinical, radiological and functional variables in two independent cohorts of ICH patients. In addition, we further studied the effect of TIMP-1 in an experimental tail-bleeding model.

## Results

### Clinical and demographic characteristics according to hematoma location

Clinical and demographic characteristics of both cohorts according to hematoma location at admission and follow-up data are shown in Tables 1 and 2.

**Table 1:** Admission demographic and clinical characteristics of the complete CHN cohort (n = 29) after dividing by hematoma location (lobar n = 16, and deep n = 13). Continuous variables are presented as median [interquartile range]. Intraventricular Hemorrhage (IVH), modified Rankin Scale (mRS), National Institute of Health Stroke Scale (NIHSS). Bold numbers indicate statistically significant p-values.

	ICH	Lobar ICH	Deep ICH	p value
	(n = 29)	(n = 16)	(n = 13)	
Age (years)	76 [66–85]	72.5 [65–87]	82.0 [68–85]	0.442
Sex (Female), n (%)	11 (37.9)	7 (43.8)	4 (30.8)	0.474
Hypertension (Yes), n (%)	15 (51.7)	6 (37.5)	9 (69.2)	0.089
Diabetes (Yes), n (%)	7 (24.1)	3 (18.8)	4 (30.8)	0.452
Dyslipidemia (Yes), n (%)	11 (37.9)	7 (43.8)	4 (30.8)	0.474
Glucose (mg/mL)	133 [115-163]	133 [110-163]	128 [117-179]	0.834
**Hematoma volume at admission (mL)**	18 [7–46]	38 [19–69]	8 [4–17]	**<0.001**
**Hematoma volume 24–48 h (mL)**	20 [5–46]	42 [23–58]	9.4 [3.6–18]	**0.004**
Hematoma growth (Yes), n (%)	3 (11.5)	1 (7.7)	2 (15.4)	0.539
IVH (Yes), n (%)	9 (31)	7 (43.8)	2 (15.4)	0.101
mRS at admission (Score)	0 [0–0]	0.0 [0–1]	0.0 [0–0])	0.162
mRS 90 days (Score)	4 [2–6]	4.0 [3–6]	4.0 [2–6]	0.534
NIHSS at admission (Score)	8 [4–17]	9.0 [3–17.7]	5.0 [4–16.5]	0.965
Mortality 90 days (Yes), n (%)	9 (33.3)	6 (42.9)	3 (23.1)	0.276
MMP-1 (ng/mL)	6.3 [3.5–12.1]	4.8 [3.3–7.56]	9.7 [4.1–13.7]	0.200
MMP-2 (ng/mL)	110.5 [85.1–144.4]	105.6 [82–134.6]	111.6 [92.8–165]	0.382
**MMP-7 (ng/mL)**	17.3 [14–22.5]	15.3 [13–19]	21.1 [15.5–24.3]	**0.048**
MMP-9 (ng/mL)	237 [137.8–338]	257.3 [115.7–334.4]	223.8 [144–388]	1
MMP-10 (pg/mL)	577.9 [439.3–855.4]	485.1 [400–742.1]	589.6 [473.6–936.4]	0.216
TIMP-1 (ng/mL)	162.3 [139.8–225.1]	166.5 [122.1–232.1]	162.3 [146.5–222.4]	0.913

**Table 2:** Admission demographic and clinical characteristics of the complete VdH cohort (n = 76) after dividing by hematoma location (lobar n = 28, and deep n = 48). Continuous variables are presented as median [interquartile range]. Intraventricular Hemorrhage (IVH), modified Rankin Scale (mRS), National Institute of Health Stroke Scale (NIHSS). Bold numbers indicate statistically significant p-values.

	ICH	Lobar ICH	Deep ICH	p value
	(n = 76)	(n = 28)	(n = 48)	
Age (years)	72 [66–80]	77 [68–82]	69 [65–77]	0.060
Sex (Female), n (%)	33 (43.4)	12(42.9)	21 (43.7)	1
**Hypertension (Yes), n (%)**	66 (86.8)	21 (75.0)	45 (93.7)	**0.048**
Diabetes (Yes), n (%)	21 (27.6)	10 (35.7)	11 (22.9)	0.348
Dyslipidemia (Yes), n (%)	46 (60.5)	19 (67.9)	27 (56.2)	0.450
**Glucose (mg/mL)**	128 [110–164]	141.5 [117–216.5]	124 [108–148]	**0.037**
**Hematoma volume at admission (mL)**	10.6 [3.9–35]	43.7 [15.5–77]	5.8 [3.2–19.3]	**<0.0001**
**Hematoma volume 24–48 h (mL)**	8 [3.6–37.1]	39 [20.2–48.1]	5.7 [3.3–14.8]	**0.001**
Hematoma growth (Yes), n (%)	12 (27.3)	3 (20.0)	9 (31.0)	0.673
IVH (Yes), n (%)	32 (42.1)	12 (42.9)	20 (41.7)	1
mRS at admission (Score)	0 [0–2]	0 [0–2]	0 [0–1.7]	0.782
mRS 90 days (Score)	4 [2–6]	5 [2–6]	3 [2–5.7]	0.198
NIHSS at admission (Score)	12 [6.7–19.2]	15 [9–21]	11 [6–19]	0.227
Mortality 90 days (Yes), n (%)	21 (31.3)	11 (45.8)	10 (23.3)	0.102
MMP-1 (ng/mL)	7.245 [3.9–11.9]	6.09 [2.9–11.5]	8.33 [5.4–12.3]	0.171
MMP-2 (ng/mL)	106.4 [83.7–116.6]	108.7 [83.7–115.3]	105.17 [83.7–117.0]	1
MMP-7 (ng/mL)	20.7 [16.0–27.6]	23.9 [16.3–31.8]	19.5 [16.0–26.4]	0.208
MMP-9 (ng/mL)	110.1 [78.3–160.4]	121.6 [78.3–205.1]	106.4 [77.6–140.4]	0.306
MMP-10 (pg/mL)	478.9 [297.7–710.4]	492.0 [290.4–963.0]	478.9 [297.7–599.2]	0.410
TIMP-1 (ng/mL)	202.9 [169.7–243.5]	225.4 [174.1–249.7]	199.2 [169.3–235.3]	0.294

In the CHN cohort (Table 1), the median [interquartile range, IQR] age was 76 [66–85] years, almost 40% were female, 52% had hypertension and the severity of the stroke was moderate (median [IQR], NIHSS = 8 [4–17]). Ninety days after ICH, the mortality rate reached 33% (n = 9) and survivors were moderate to severely disabled (mRS = 4 [2–6]). Patients with lobar ICH (n = 16, 55%) had larger hematoma volumes at admission when compared to deep location (38 [19–69] mL lobar vs. 8 [4–17] mL deep, p < 0.001), and 24–48 hours after ICH onset (42 [23–58] mL lobar vs. 9.4 [3.6–18] mL deep, p = 0.004). There was a tendency to a higher prevalence of hypertension (dichotomized) in patients with deep hematoma (p = 0.089). Only MMP-7 levels were higher in patients with deep hematoma when compared to lobar (median [IQR], 21.1 [15.5–24.3] ng/mL deep vs. 15.3 [13.0–19.0] ng/mL lobar, p = 0.048). We did not find differences on functional outcomes, mortality or the rest of MMPs and TIMP-1 between these two ICH locations (Table 1).

In the VdH cohort (Table 2), the median [IQR] age of ICH patients was 72 [66–80] years, nearly 44% were female, almost 90% presented hypertension and stroke severity ranged from moderate to severe (median [IQR], NIHSS = 12 [6.7–19.2]). Ninety days after ICH, mortality rate was 31% (n = 21) and survivors presented moderate to severe disability (mRS = 4 [2–6]). ICH location was lobar in 28 patients (37%) and deep in 48 (63%). As reported for the CHN cohort, hemorrhage volumes were also larger in patients with lobar hematoma when compared to deep location at admission (43.7 [15.55–77] mL lobar vs.5.8 [3.25–19.27] mL deep, p = 0.0001) and 24–48 hours after ICH (39 [20.2–48.11] mL lobar vs. 5.7 [3.3–14.8] mL deep, p = 0.0015). Furthermore, the prevalence of hypertension was higher in patients with deep hematoma when compared to lobar (94% deep vs. 75% lobar, p = 0.047), while glucose levels were lower (124 [108–147.75] deep vs. 141.5 [116.75–216.5] mg/mL lobar, p = 0.036). No significant association of hematoma location was observed with any of the functional outcomes, mortality, MMPs or TIMP-1 (Table 2).

### Association of MMPs and TIMP-1 with clinical and radiological variables

We performed a univariate analysis to assess the relationship between plasma MMPs and TIMP-1 and clinical and radiological variables (Tables 3 and 4).

**Table 3:** Association of admission hematoma volume with clinical parameters and MMPs and TIMP-1 levels in the CHN cohort (n = 29). Intraventricular Hemorrhage (IVH), modified Rankin Scale (mRS), National Institute of Health Stroke Scale (NIHSS). Bold numbers indicate statistically significant p-values.

	B (95% CI)	Beta	p value
Age (years)	0.52 (−0.87–1.90)	0.15	0.450
Sex (Female)	−6.1 (−40.7–28.5)	−0.07	0.721
**Hypertension**	**−32.2 (−63.4–0.94)**	**−0.38**	**0.044**
Diabetes	−28.1 (−65.9–9.7)	−0.28	0.139
Dyslipidemia	−22.9 (−56.4–10.7)	−0.26	0.173
Glucose (mg/mL)	0.22 (−0.17–0.60)	0.22	0.262
**Hematoma location (Lobar)**	**43.7 (14.5–72.8)**	**0.51**	**0.005**
Hematoma growth	−9.1 (−32.4–14.2)	−0.16	0.426
**IVH**	**44.2 (12.2–76.2)**	**0.48**	**0.008**
mRS at admission (Score)	−11.9 (−40.6–16.6)	−0.16	0.398
mRS 90 days (Score)	**8.0 (−0.23–16.2)**	**0.37**	**0.056**
**NIHSS at admission (Score)**	**2.8 (1.14–4.41)**	**0.56**	**0.002**
**Mortality 90 days**	**46.0 (12.5–79.5)**	**0.49**	**0.009**
MMP-1 (ng/mL)	0.31 (−1.06–1.70)	0.10	0.639
MMP-2 (ng/mL)	0.15 (−0.23–0.53)	0.16	0.419
MMP-7 (ng/mL)	−1.69 (−3.80–0.42)	−0.32	0.112
MMP-9 (ng/mL)	−0.01 (−0.14–0.13)	−0.02	0.934
MMP-10 (pg/mL)	0.02 (−0.03–0.07)	0.14	0.480
**TIMP-1 (ng/mL)**	**0.19 (0.09–0.28)**	**0.60**	**0.001**

**Table 4:** Association of admission hematoma volume with clinical parameters and MMPs and TIMP-1 levels in the VdH cohort (n = 76). Intraventricular Hemorrhage (IVH) modified Rankin Scale (mRS), National Institute of Health Stroke Scale (NIHSS). Bold numbers indicate statistically significant p-values.

	B (95% CI)	Beta	p value
**Age (years)**	**1.33 (0.4–2.27)**	**0.32**	**0.007**
Sex (Female)	−3.24 (−22.02–15.53)	−0.04	0.736
**Hypertension**	**−28.94 (−55.1–2.78)**	**−0.25**	**0.033**
Diabetes	5.12 (−15.36–25.61)	0.06	0.625
Dyslipidemia	−14.28 (−32.97–4.41)	−0.18	0.139
**Glucose (mg/mL)**	**0.25 (0.1–0.41)**	**0.35**	**0.002**
**Hematoma location (Lobar)**	**46.22 (29.87–62.58)**	**0.55**	**<0.001**
Hematoma growth	−12.87 (−26.32–0.58)	−0.28	0.068
IVH	17.7 (−0.64–36.03)	**0.22**	**0.063**
mRS at admission (Score)	6.11 (−0.9–13.12)	0.20	**0.092**
**mRS 90 days (Score)**	**10.9 (6.01–15.79)**	**0.50**	**<0.001**
**NIHSS at admission (Score)**	**2.05 (0.99–3.11)**	**0.41**	**<0.001**
**Mortality 90 days**	**48.72 (29.43–68.02)**	**0.53**	**<0.001**
MMP-1 (ng/mL)	0.45 (−0.96–1.86)	0.07	0.534
MMP-2 (ng/mL)	0.15 (−0.15–0.46)	0.12	0.324
**MMP-7 (ng/mL)**	**0.83 (0.32–1.34)**	**0.36**	**0.002**
MMP-9 (ng/mL)	0.05 (−0.04–0.14)	0.12	0.308
MMP-10 (pg/mL)	0.01 (−0.01–0.03)	0.14	0.236
**TIMP-1 (ng/mL)**	**0.17 (0.03–0.32)**	**0.27**	**0.022**

First in the CHN cohort, the MMPs studied were not associated with either cardiovascular risk factors, functional outcomes or mortality (Supplemental Table 1). However, TIMP-1 levels were positively associated with admission hematoma volume (0.19; 95% CI 0.09 to 0.28 p = 0.001, Table 3). We also assessed the association between admission hematoma volume and different clinical and radiological parameters. Admission NIHSS (2.8; 95% CI 1.1 to 4.4, p = 0.002), 90-days mortality (46; 95% CI 12.5 to 79.5, p = 0.009) and ventricle’s blood (44.2; 95% CI 12.2 to 76.2, p = 0.008) were positively associated with admission hematoma volume. Moreover, patients with a lobar location had a significantly higher hemorrhage volume than patients with a deep location (43.7; 95% CI 14.5 to 72.8, p = 0.005). Hypertensive patients showed lower hematoma volume (−32.2; 95% CI -63.4 to −0.94, p = 0.044, Table 3).

In the VdH cohort, MMP-2 levels were associated with age (0.95; 95% CI 0.27 to 1.63, p = 0.007), mRS 90 days (3.8; 95% CI 0.48 to7.07, p = 0.025), hypertension and 90-days mortality (OR = 1.032, 95% CI 1.002 to 1.064, p = 0.039 and OR = 1.025, 95% CI 1.003 to 1.048, p = 0.027, respectively). MMP-10 levels were associated with age (11.7; 95% CI 1.4 to 21.9, p = 0.025) and diabetes (OR = 1.001, 95% CI 1.000 to 1.003, p = 0.021), and MMP-9 levels were lower in female patients (−59.9; 95% CI −98.1 to −7.7, p = 0.022, Supplemental Table 2). Moreover, we again observed that admission hematoma volume was positively associated with TIMP-1 levels (0.17; 95% CI 0.03 to 0.32, p = 0.022, Table 4) but also with MMP-7 levels (0.83; 95% CI 0.32 to 1.34, p = 0.002, Table 4). Regarding the association between admission hematoma volume and other clinical and radiological parameters, we found a positive association of this variable, with glucose levels (0.25; 95% CI 0.1 to 0.41 p = 0.0021), age (1.3; 95% CI 0.4 to 2.3, p = 0.007), mortality (48.7; 95% CI 29.4 to 68.0, p < 0.001) and with admission NIHSS (2.05; 95% CI 0.99 to 3.11, p < 0.001). Again, patients, with lobar location had a significantly higher hemorrhage volume than patients with a deep location (46.2; 95% CI 29.9 to 62.6, p < 0.001). Hypertensive patients showed lower hematoma volume (−28.94; 95% CI −55.1 to −2.78, p = 0.033, Table 4).

### TIMP-1 as a potential biomarker for hematoma volume

We aimed to further analyze the association of plasmatic TIMP-1 with the volume of hemorrhage at admission observed in both cohorts by multivariate regression analysis.

First, in the CHN cohort, the multivariate analysis showed a significant association of TIMP-1 and hemorrhage volume after adjusting for confounding factors in all tested models (models 1 to 3, Table 5). This association was further confirmed in the VdH cohort in both, the univariate and the multivariate analysis (Table 6).

**Table 5:** Multivariate linear regression analysis for admission hematoma volume (dependent variable) in the CHN cohort. Independent variables, model 1 includes: TIMP-1, hematoma location, and hypertension (HTA); model 2: TIMP-1, hematoma location and NIHSS; and model 3: TIMP-1, hematoma location, hypertension (HTA) and NIHSS.

	Model 1 (TIMP-1, hematoma location and HTA)		Model 2 (TIMP-1, hematoma location and NIHSS)		Model 3 (TIMP-1, hematoma location, HTA and NIHSS)	
	B (95% CI)	p value	B (95% CI)	p value	B (95% CI)	p value
TIMP-1	0.16 (0.08–0.25)	0.001	0.13 (0.04–0.21)	0.004	0.13 (0.04–0.21)	0.004
Location (lobar)	30.37 (5.9–54.9)	0.017	34.48 (13.3–55.7)	0.003	32.8 (10.1–55.6)	0.007
HTA	−19.38 (−43.5–4.7)	0.111			−5.8 (−31.1–19.5)	0.641
NIHSS			1.85 (0.56–3.15)	0.007	1.7 (0.19–3.19)	0.029

**Table 6:** Multivariate linear regression analysis for admission hematoma volume (dependent variable) in the VdH cohort. Independent variables, model 1 includes: TIMP-1, hematoma location, and hypertension (HTA); model 2: TIMP-1, hematoma location, NIHSS; and model 3: TIMP-1, hematoma location, hypertension (HTA), and NIHSS.

	Model 1 (TIMP-1, hematoma location and HTA)		Model 2 (TIMP-1, hematoma location and NIHSS)		Model 3 (TIMP-1, hematoma location, HTA and NIHSS)	
	B (95% CI)	p value	B (95% CI)	p value	B (95% CI)	p value
TIMP-1	0.15 (0.03–0.27)	0.018	0.17 (0.06–0.28)	0.003	0.17 (0.06–0.28)	0.003
Location (lobar)	42.03 (25.36–58.69)	<0.001	42.15 (28.18–56.13)	<0.001	41.24 (26.52–55.96)	<0.001
HTA	−12.58 (−35.53–10.38)	0.287			−4.34 (−24.92–16.24)	0.681
NIHSS			1.99 (1.16–2.82)	<0.001	1.96 (1.11–2.81)	<0.001

**Figure 1:**
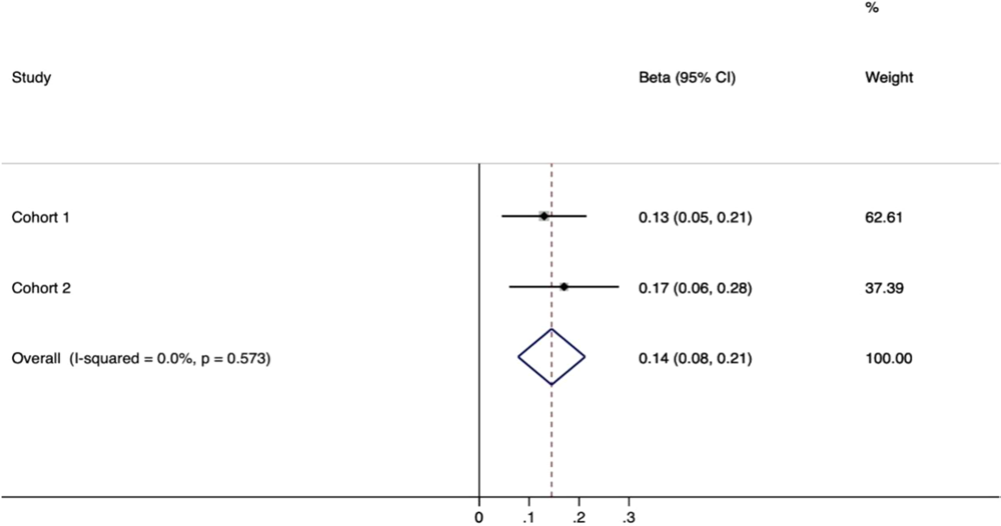
Fixed effect meta-analysis to assess the effect of admission TIMP-1 and hematoma volume in ICH patients. Beta coefficients were adjusted for location, hypertension and NIHSS in both cohorts (Cohort 1 = CHN and Cohort 2 = VdH).

When we combined the results from the two cohorts, we observed a statistically significant association between TIMP-1 and hematoma volume (Overall β = 0.14, 95% CI = 0.08–0.21, Fig. 1). These results suggest that TIMP-1 might be a potential biomarker for ICH.

### TIMP-1 effects in experimental haemorrhage

**Figure 2:**
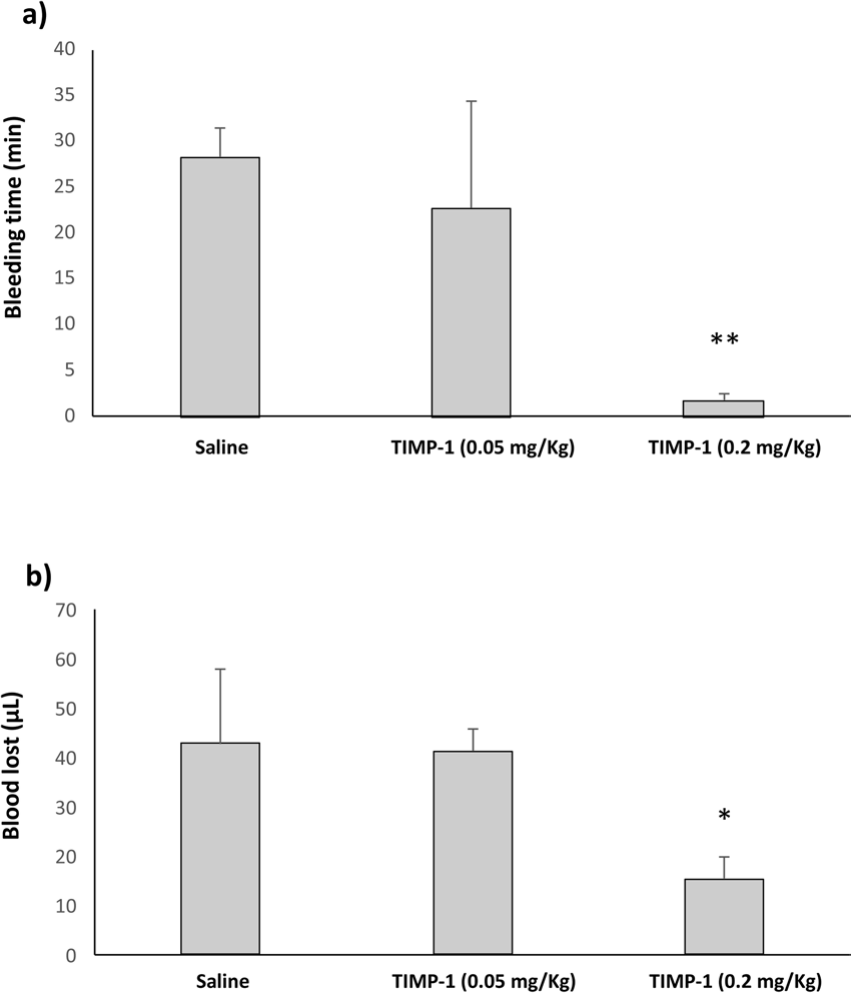
TIMP-1 effects in mice experimental tail bleeding. (**a**) Bleeding time of animals treated with saline (n = 5), TIMP-1 (0.05 mg/Kg, n = 4) and TIMP-1 (0.2 mg/Kg, n = 3). (**b**) Blood lost obtained after experimental bleeding model. **p < 0.01 and *p < 0.05 vs. Saline.

After finding an association between hematoma volume and TIMP-1, we further explored the effects of TIMP-1 in an experimental model of tPA-induced haemorrhage. Our results showed that an experimental dose of TIMP-1 (0.05 mg/Kg) did not decrease the maximum bleeding time allowed (30 minutes). However, mice injected with the highest dose of TIMP-1 (0.2 mg/Kg), showed a much shorter bleeding time (1.8 ± 0.7 vs. 28.3 ± 3.3 min, p < 0.01, Fig. 2a) and blood lost (15.3 ± 4.7 vs. 42.8 ± 14.9 μL, p < 0.05, Fig. 2b). These results suggest that TIMP-1 might be able to control experimental haemorrhage.

## Discussion

The main objective of this study was to explore the relationship of MMPs and TIMP-1 with clinical, radiological and functional variables in two independent cohorts of ICH patients. Hematoma volume and expansion have been associated with poor prognosis, neurological detriment and disability after ICH^21^. We reported for the first time that circulating levels of TIMP-1 at admission are associated with hematoma volume independently of hypertension, NIHSS and hematoma location in two independent cohorts of ICH patients.

TIMP-1 is an endogenous inhibitor of MMPs present under normal conditions in tissues to regulate MMPs activity^22^. Studies addressing the role of TIMP1 in ICH are scarce. In this study, we report that TIMP-1 is significantly associated with hematoma volume in two independent cohorts, and after a combined meta-analysis, suggesting that TIMP-1 might be potential critical biomarker in ICH. These results are in agreement with other studies showing increased concentrations of TIMP-1 during the acute phase of ICH, that remained highly sustained during the first week after stroke onset^23^, and showing TIMP-1 association with early mortality rates^24^, supporting a potential role of TIMP-1 as severity biomarker in ICH. The elevated secretion of TIMP-1 might be induced to lessen MMPs activity and thus, to prevent further brain damage^25^. However, it might not be enough to counterbalance the concomitant upregulation of MMPs. Likewise, it has been reported a relative increase in MMP-9/TIMP-1 ratio associated with symptomatic ICH^26^ and Subarachnoid Haemorrhage (SAH)^27^ supporting the hypothesis that perturbations in MMPs/TIMPs levels may contribute to the matrix disruption associated with cerebral aneurysms^28^.

Additionally, we found that administration of TIMP-1 (approximately 10-fold circulating levels) is able to control experimental acute bleeding in mice. These findings are consistent with the overall idea that in conditions of brain haemorrhage, damage, and inflammation, there may be an imbalance in the dynamic equilibrium between MMPs and TIMPs, so that any way of enhancing the inhibitor side of the equation might be beneficial^29,30^. Supporting this hypothesis, previous data in TIMP-1 knockout mice showed exacerbated BBB disruption and brain injury^31^, while adenoviral TIMP-1 overexpression in mice, reduced brain lesion volume, neuronal damage and BBB leakage after ischemia^32^. Moreover, MMP inhibitors significantly decreased bleeding and brain damage in experimental models of haemorrhage, including ICH^33,34^.

One of the main factors that influences ICH outcome is the hematoma location; deep and lobar predominantly^35^ that usually results in different hematoma volumes. Despite the relevance of this parameter for ICH outcome, there are few clinical data regarding the association of MMPs with hematoma location. Abilleira *et al*. reported higher MMP-9 plasmatic levels in patients with deep-associated ICH^36^. Moreover, in cerebral amyloid ICH, typically located in lobar regions, MMP-2 and -9 were up-regulated in perihematomal area of the brains^20,37^. In our study, CHN patients with deep hematoma location showed elevated plasmatic MMP-7 levels compared to patients with lobar hematoma. MMP-7/matrilysin is a small, secreted metalloproteinase capable of digesting a large series of proteins of the extracellular matrix^38^. A recent population-based cohort study followed for more than 10 years, reported that high serum levels of MMP-7 were independently associated with increased risk of incident spontaneous SAH suggesting its possible use as a predictive marker for SAH^39^. Likewise, several studies with MMP-7 null mice indicated that the robust expression of MMP-7 during severe inflammation lead to irreparable tissue damage^40^. Furthermore, we observed an association of this metalloprotease with hematoma volume in the VdH cohort. Therefore, our results suggest that MMP-7 might be involved in the pathophysiology of deep-ICH although it may be partly dependent on risk factors, therefore, further studies must be performed to confirm these data.

Other associations found in this study, such as MMP-10 with age and diabetes and MMP-2 with age, hypertension and 90-days mortality, were only significant in VdH possibly explained by larger sample size and statistical power of this cohort. However, these associations were lost after adjusting for risk factors of ICH. Previous data about the association of high MMP-10 levels with microvascular complications in diabetes type 1 in clinical and experimental studies^41,42^ could explain the association found in ICH patients. Regarding MMP-2, several studies support the association of MMP-2 with hypertension and mortality in diabetic patients and in patients with heart failure^43,44^. However, concerning spontaneous ICH patients, elevated levels of MMP-2 were only found at admission decreasing during the first 24 hours of hospitalization^23^ and, specifically at tissue level in β-amyloid-damaged vessels located far from the acute ICH^20^. Therefore, future studies with larger sample size might confirm these data.

Our work has several limitations: first the small sample sizes of our series make our results to be interpreted carefully. Therefore, data from this study should be confirmed in larger cohorts before definitive clinical conclusions can be drawn. The observational study design did not allow us to establish a cause-effect relationship between TIMP-1 and hematoma volume, although the effect of TIMP-1 reducing bleeding time in an experimental model of tail bleeding supports the hypothesis that TIMP-1 could be a promising strategy to control hematoma volume in ICH. However, further experimental models of ICH using deficient mice might be performed.

## Conclusion

We report for the first time that circulating TIMP-1 is associated with hematoma volume in two independent ICH cohorts and when we combined the results from both cohorts suggesting the potential of TIMP-1 as biomarker for ICH. Additionally, TIMP-1 administration significantly reduces experimental bleeding. Therefore, it could be considered that after ICH increased TIMP-1 levels might not be sufficient to counterbalance the upregulation of MMPs, and that its exogenous administration could be a beneficial strategy for ICH in order to reduce MMPs activity and restore the MMPs/TIMPs balance.

## Materials and methods

### Study populations

Spontaneous non‐traumatic ICH patients were recruited from two different Spanish tertiary hospitals (Tables 1 and 2): a cohort from Complejo Hospitalario de Navarra (CHN, n = 29), collected between February 2016 and June 2017; and a larger cohort of patients (n = 76) from Hospital Universitario Vall d’Hebron (VdH) collected between August 2012 and December 2013. These prospective studies were approved by the ethics committee of the Navarra Government (63/2015) and the Catalonia Government [PR(AG)157–2011] respectively. All patients or their “legally authorised representative” provided written informed consent according to Helsinki declaration. The study included only patients with spontaneous nontraumatic supratentorial intracerebral hemorrhage (ICH), diagnosed by experienced neurologists of the Stroke Unit by initial cranial non-contrast computed tomography (CT) at admission in the emergency department. Patients with brainstem/infratentorial ICH and/or secondary ICH (vascular malformation, aneurysm, tumors, ischemic stroke with haemorrhagic transformation) were excluded. We excluded other causes of intracranial haemorrhages, as subdural hematoma, epidural bleeds, and subarachnoid hemorrhage. However, we included patients who had suffered subarachnoid and intraventricular haemorrhage (IVH), although always associated with concurrent parenchymal hematoma. Systemic cancer and inflammatory systemic condition patients were also excluded from the study. ICH was classified by its location by experienced neurologist as previously reported by Aguilar I *et al*.^45^. Demographics and previous antiplatelet and/or anticoagulation therapy were registered. Reversal of anticoagulation was indicated in some patients according to current guidelines.

### Radiologic measurements

The primary radiologic outcomes were hemorrhage volume (admission volume and after the following 24–48 hours), hematoma location, hematoma expansion (more than 6 mL or 33% growth during the first 24 hours) and IVH. Hematoma volume was quantified by using the ABC/2 method^46,47^. This well-validated method, consist on measuring the greatest axial dimension of the hematoma (A), a perpendicular diameter on the same slice (B), and the cranio-caudal dimension (C) estimated by counting the number of slices where the hematoma is present and multiplying that number by the slice thickness. The hematoma volume is estimated by multiplying these three dimensions together and dividing by 2, which estimates the simplified volume of an ellipse.

### Neurological and functional outcomes

Clinical severity was assessed at admission using the National Institute of Health Stroke Scale (NIHSS)^48^ and initial prognosis was evaluated by ICH score^49^. Functional outcome was defined by modified Rankin Scale (mRS)^50^ and mortality rates at 90 days after onset to evaluate the mid-term outcome of patients. Previous functional situation evaluated by mRS was registered at admission.

### Sample collection

Citrated plasma samples of venous blood were obtained from both study cohorts at the moment of hospitalization (admission). All samples were processed within 4 hours of collection and stored at −80 °C. Samples were thawed on ice and thoroughly vortexed prior to beginning any experiment. Plasma MMP-1, −2, −7, −9, −10 were measured with MAP Human MMP panel 2 (Milliplex, Millipore, Darmstadt, Germany) in a Luminex 200 platform (Luminex technology, Comercial Rafer S.L., Spain) and TIMP-1 was measured with ELISA kit for TIMP-1 (R&D Systems, Minneapolis, US) in an automated ELISA system (Triturus, Movaco, Spain). All experiments were performed according to manufacturer´s instructions by blinded investigators.

### Bleeding assay

Bleeding assay was performed in 5-week-old male C57/BL6J mice (n = 12, Envigo, Spain). All experiments in this study were conducted according to the European Community guidelines for the ethical animal care and use of laboratory animals (2010/63/EU) and approved by the University of Navarra Animal Research Review Committee (ref. ^46,12^). The experimental protocol is shown in Supplemental Fig. 1. Briefly, animals were anesthetized with 2.5% isoflurane, placed in prone position and tPA (0.05 mg/Kg) was injected through the ocular plexus. Five minutes later, TIMP-1 (0.05 and 0.2 mg/Kg) or saline were injected through the femoral vein catheter (10% bolus and 90% perfusion during 40 minutes). A distal 5 mm segment of the tail was transected with a scalpel blade 5 minutes after starting the treatments. The tail was immediately immersed in a tube containing pre-warmed saline (37 °C in a water bath). Bleeding time was defined as the time elapsed until bleeding stops for a maximum of 30 min. Blood lost was defined as the amount of blood collected during 30 min and absorbance was measured in a spectrophotometer (Tecan Sunrise, Switzerland) and quantified against a standard curve generated with known quantities of blood^33^.

### Statistical analysis

Descriptive statistics (median, interquartile range, frequency and percentage) were defined for all clinical, radiological and functional variables. Normality was assessed using the Shapiro-Wilk test. Bivariate comparisons of characteristics at admission between ICH location (deep and lobar index ICH) were performed using Student t test or Mann-Whitney U test for continuous variables, and χ2 test or Fisher exact test for categorical variables. All covariates associated with the end point in the univariate analysis, considering a threshold of p < 0.1 in both cohorts, were included in the multivariate linear regression analyses for the target molecules of the study (MMPs and TIMP-1). Due to the small sample size in one of the cohorts, the following baseline models were considered for adjustment: model 1, TIMP-1, location, and hypertension (yes/no); and model 2, TIMP-1, location and NIHSS. Finally, we conducted a fixed effect meta-analysis to combine the data from our two cohorts in a meta-analysis. Statistical analysis of the data from experimental model was done using Mann-Whitney U two-tailed test.

## Supplementary information

Supplementary information.

## Data Availability

The datasets generated during and/or analysed during the current study are available from the corresponding author on reasonable request.

## References

[1] Dichgans, M., Pulit, S. L. & Rosand, J. Stroke genetics: discovery, biology, and clinical applications. *The Lancet Neurology*. 10.1016/S1474-4422(19)30043-2 (2019). 10.1016/S1474-4422(19)30043-2 30975520

[2] Al-Shahi Salman, R. *et al*. Absolute risk and predictors of the growth of acute spontaneous intracerebral haemorrhage: a systematic review and meta-analysis of individual patient data. *Lancet Neurol*. 10.1016/S1474-4422(18)30253-9 (2018). 10.1016/S1474-4422(18)30253-9 PMC6143589 30120039

[3] Selim, M. Unmet needs and challenges in clinical research of intracerebral hemorrhage. *Stroke*. 10.1161/STROKEAHA.117.019541 (2018). 10.1161/STROKEAHA.117.019541 PMC5916316 29618558

[4] Hanley, D. F. *et al*. Efficacy and safety of minimally invasive surgery with thrombolysis in intracerebral haemorrhage evacuation (MISTIE III): a randomised, controlled, open-label, blinded endpoint phase 3 trial. *Lancet*. 10.1016/S0140-6736(19)30195-3 (2019). 10.1016/S0140-6736(19)30195-3 PMC6894906 30739747

[5] Ziai, W. C. *et al*. A randomized 500-subject open-label phase 3 clinical trial of minimally invasive surgery plus alteplase in intracerebral hemorrhage evacuation (MISTIE III). *Int. J. Stroke*. 10.1177/1747493019839280 (2019). 10.1177/1747493019839280 PMC6706298 30943878

[6] Urday, S. *et al*. Targeting secondary injury in intracerebral haemorrhage-perihaematomal oedema. *Nature Reviews Neurology*. 10.1038/nrneurol.2014.264 (2015). 10.1038/nrneurol.2014.264 25623787

[7] Biffi, A. *et al*. Association between blood pressure control and risk of recurrent intracerebral hemorrhage. *JAMA - J. Am. Med. Assoc*. 10.1001/jama.2015.10082 (2015). 10.1001/jama.2015.10082 PMC4737594 26325559

[8] Alg, V. S. *et al*. Association of functional MMP-2 gene variant with intracranial aneurysms: case-control genetic association study and meta-analysis. *Br. J. Neurosurg*. 10.1080/02688697.2018.1427213 (2018). 10.1080/02688697.2018.1427213 29334797

[9] Zhang, X. *et al*. Matrix metalloproteases-mediated cleavage on β-dystroglycan may play a key role in the blood–brain barrier after intracerebral hemorrhage in rats. *Med. Sci. Monit*. 10.12659/MSM.908500 (2019). 10.12659/MSM.908500 PMC6362757 30686819

[10] Purroy, A. *et al*. Matrix metalloproteinase-10 deficiency delays atherosclerosis progression and plaque calcification. *Atherosclerosis*. 10.1016/j.atherosclerosis.2018.09.022 (2018). 10.1016/j.atherosclerosis.2018.09.022 30268068

[11] Duits, F. H. *et al*. Matrix Metalloproteinases in Alzheimer’s Disease and Concurrent Cerebral Microbleeds. *J. Alzheimer’s Dis*. 10.3233/JAD-143186 (2015). 10.3233/JAD-143186 26402072

[12] Brew, K. & Nagase, H. The tissue inhibitors of metalloproteinases (TIMPs): An ancient family with structural and functional diversity. *Biochimica et Biophysica Acta - Molecular Cell Research*. 10.1016/j.bbamcr.2010.01.003 (2010). 10.1016/j.bbamcr.2010.01.003 PMC2853873 20080133

[13] Yang, Y. & Rosenberg, G. A. Matrix metalloproteinases as therapeutic targets for stroke. *Brain Research*. 10.1016/j.brainres.2015.04.024 (2015). 10.1016/j.brainres.2015.04.024 PMC4569515 25916577

[14] Howe, M. D. *et al*. Serum markers of blood-brain barrier remodeling and fibrosis as predictors of etiology and clinicoradiologic outcome in intracerebral hemorrhage. *Front. Neurol*. 10.3389/fneur.2018.00746 (2018). 10.3389/fneur.2018.00746 PMC6143812 30258397

[15] Li, N. *et al*. Association of molecular markers with perihematomal edema and clinical outcome in intracerebral hemorrhage. *Stroke*. 10.1161/STROKEAHA.112.673590 (2013). 10.1161/STROKEAHA.112.673590 23391772

[16] Florczak-Rzepka, M., Grond-Ginsbach, C., Montaner, J. & Steiner, T. Matrix metalloproteinases in human spontaneous intracerebral hemorrhage: An update. *Cerebrovascular Diseases*. 10.1159/000341686 (2012). 10.1159/000341686 23052179

[17] Lorente, L. *et al*. Association between serum tissue inhibitor of matrix metalloproteinase-1 levels and mortality in patients with severe brain trauma injury. *PLoS One*. 10.1371/journal.pone.0094370 (2014). 10.1371/journal.pone.0094370 PMC3984169 24728097

[18] Lorente, L. *et al*. Persistently high circulating tissue inhibitor of matrix metalloproteinase-1 levels in non-survivor brain trauma injury patients. *J. Crit. Care*. 10.1016/j.jcrc.2019.02.014 (2019). 10.1016/j.jcrc.2019.02.014 30802757

[19] Lorente, L. *et al*. Serum tissue inhibitor of matrix metalloproteinase-1 levels are associated with mortality in patients with malignant middle cerebral artery infarction. *BMC Neurol*. 10.1186/s12883-015-0364-7 (2015). 10.1186/s12883-015-0364-7 PMC4499187 26162891

[20] Hernandez-Guillamon, M. *et al*. MMP-2/MMP-9 plasma level and brain expression in cerebral amyloid angiopathy-associated hemorrhagic stroke. *Brain Pathol*. 10.1111/j.1750-3639.2011.00512.x (2012). 10.1111/j.1750-3639.2011.00512.x PMC8029059 21707819

[21] Haller, J. T., Wiss, A. L., May, C. C., Jones, G. M. & Smetana, K. S. Acute Management of Hypertension Following Intracerebral Hemorrhage. *Critical Care Nursing Quarterly*. 10.1097/CNQ.0000000000000247 (2019). 10.1097/CNQ.0000000000000247 30807338

[22] Cunningham, L. A., Wetzel, M. & Rosenberg, G. A. Multiple roles for MMPs and TIMPs in cerebral ischemia. *GLIA*. 10.1002/glia.20169 (2005). 10.1002/glia.20169 15846802

[23] Alvarez-Sabín, J. *et al*. Temporal profile of matrix metalloproteinases and their inhibitors after spontaneous intracerebral hemorrhage: Relationship to clinical and radiological outcome. *Stroke*. 10.1161/01.STR.0000126827.69286.90 (2004). 10.1161/01.STR.0000126827.69286.90 15087562

[24] Lorente, L. *et al*. High Serum Tissue Inhibitor of Matrix Metalloproteinase-1 Levels and Mortality in Patients with Spontaneous Intracerebral Hemorrhage. *World Neurosurg*. 10.1016/j.wneu.2019.10.106 (2020). 10.1016/j.wneu.2019.10.106 31669537

[25] Zhang, J. X., Zhang, Z. Y. & Cheng, Y. Elevated MMP-1 and TIMP-1 are related with acute cerebral infarction patients with diabetes mellitus. *Int. J. Clin. Exp. Med*. **10**(12) 16555–16561 (2017).

[26] Piccardi, B. *et al*. Unbalanced metalloproteinase-9 and tissue inhibitors of metalloproteinases ratios predict hemorrhagic transformation of lesion in ischemic stroke patients treated with thrombolysis: Results from the MAGIC study. *Front. Neurol*. 10.3389/fneur.2015.00121 (2015). 10.3389/fneur.2015.00121 PMC4445323 26074872

[27] Fischer, M. *et al*. Differential Regulation of Matrix-Metalloproteinases and Their Tissue Inhibitors in Patients with Aneurysmal Subarachnoid Hemorrhage. *PLoS One*. 10.1371/journal.pone.0059952 (2013). 10.1371/journal.pone.0059952 PMC3610709 23555845

[28] Kim, S. C. *et al*. Matrix metalloproteinase-9 in cerebral aneurysms. *Neurosurgery*. 10.1097/00006123-199709000-00027 (1997). 10.1097/00006123-199709000-00027 9310982

[29] Sellner, J. & Leib, S. L. In bacterial meningitis cortical brain damage is associated with changes in parenchymal MMP-9/TIMP-1 ratio and increased collagen type IV degradation. *Neurobiol. Dis*. 10.1016/j.nbd.2005.09.007 (2006). 10.1016/j.nbd.2005.09.007 16257222

[30] Waubant, E. *et al*. IFNβ lowers MMP-9/TIMP-1 ratio, which predicts new enhancing lesions in patients with SPMS. *Neurology*. 10.1212/WNL.60.1.52 (2003). 10.1212/wnl.60.1.52 12525717

[31] Fujimoto, M. *et al*. Tissue inhibitor of metalloproteinases protect blood-brain barrier disruption in focal cerebral ischemia. *J. Cereb. Blood Flow Metab*. 10.1038/jcbfm.2008.59 (2008). 10.1038/jcbfm.2008.59 18560439

[32] Magnoni, S. *et al*. Neuroprotective effect of adenoviral-mediated gene transfer of TIMP-1 and -2 in ischemic brain injury. *Gene Ther*. 10.1038/sj.gt.3302894 (2007). 10.1038/sj.gt.3302894 17235293

[33] Orbe, J. *et al*. Design, synthesis, and biological evaluation of novel matrix metalloproteinase inhibitors as potent antihemorrhagic agents: From hit identification to an optimized lead. *J. Med. Chem*. 10.1021/jm501940y (2015). 10.1021/jm501940y 25686153

[34] Rodríguez, J. A. *et al*. CM352 reduces brain damage and improves functional recovery in a rat model of intracerebral hemorrhage. *J. Am. Heart Assoc*. 10.1161/JAHA.117.006042 (2017). 10.1161/JAHA.117.006042 PMC5669199 28572282

[35] Ironside, N. *et al*. Location-specific differences in hematoma volume predict outcomes in patients with spontaneous intracerebral hemorrhage. *Int. J. Stroke*. 10.1177/1747493019830589 (2020). 10.1177/1747493019830589 30747614

[36] Abilleira, S. *et al*. Matrix metalloproteinase-9 concentration after spontaneous intracerebral hemorrhage. *J. Neurosurg*. 10.3171/jns.2003.99.1.0065 (2003). 10.3171/jns.2003.99.1.0065 12854746

[37] Zhao, L. *et al*. Matrix metalloproteinase 9-mediated intracerebral hemorrhage induced by cerebral amyloid angiopathy. *Neurobiol. Aging*. 10.1016/j.neurobiolaging.2015.07.016 (2015). 10.1016/j.neurobiolaging.2015.07.016 PMC4609585 26248866

[38] Barrett, A. J., Rawlings, N. D. & Woessner, J. F. Handbook of Proteolytic Enzymes: Second Edition. Handbook of Proteolytic Enzymes: Second Edition. 10.1016/C2009-0-03628-9 (2004).

[39] Söderholm, M., Nordin Fredrikson, G., Nilsson, J. & Engström, G. High Serum Level of Matrix Metalloproteinase-7 Is Associated with Increased Risk of Spontaneous Subarachnoid Hemorrhage. *Stroke*. 10.1161/STROKEAHA.118.020660 (2018). 10.1161/STROKEAHA.118.020660 29880550

[40] Li, Q., Park, P. W., Wilson, C. L. & Parks, W. C. Matrilysin shedding of syndecan-1 regulates chemokine mobilization and transepithelial efflux of neutrophils in acute lung injury. *Cell*. 10.1016/S0092-8674(02)01079-6 (2002). 10.1016/s0092-8674(02)01079-6 12464176

[41] Toni, M. *et al*. Matrix metalloproteinase-10 plays an active role in microvascular complications in type 1 diabetic patients. *Diabetologia*. 10.1007/s00125-013-3052-4 (2013). 10.1007/s00125-013-3052-4 24078057

[42] Peeters, S. A. *et al*. Plasma levels of matrix metalloproteinase-2, -3, -10, and tissue inhibitor of metalloproteinase-1 are associated with vascular complications in patients with type 1 diabetes: The EURODIAB Prospective Complications Study. *Cardiovasc. Diabetol*. 10.1186/s12933-015-0195-2 (2015). 10.1186/s12933-015-0195-2 PMC4355971 25848912

[43] Peeters, S. A. *et al*. Plasma matrix metalloproteinases are associated with incident cardiovascular disease and all-cause mortality in patients with type 1 diabetes: A 12-year follow-up study. *Cardiovasc. Diabetol*. 10.1186/s12933-017-0539-1 (2017). 10.1186/s12933-017-0539-1 PMC5405549 28446168

[44] George, J. *et al*. Circulating matrix metalloproteinase-2 but not matrix metalloproteinase-3, matrix metalloproteinase-9, or tissue inhibitor of metalloproteinase-1 predicts outcome in patients with congestive heart failure. *Am. Heart J*. 10.1016/j.ahj.2004.11.016 (2005). 10.1016/j.ahj.2004.11.016 16169329

[45] Aguilar, M. I. & Brott, T. G. Update in Intracerebral Hemorrhage. *The Neurohospitalist*. 10.1177/1941875211409050 (2011). 10.1177/1941875211409050 PMC3726132 23983850

[46] Huttner, H. B. *et al*. Comparison of ABC/2 estimation technique to computer-assisted planimetric analysis in warfarin-related intracerebral parenchymal hemorrhage. *Stroke*. 10.1161/01.STR.0000198806.67472.5c (2006). 10.1161/01.STR.0000198806.67472.5c 16373654

[47] Kranz, P. G., Malinzak, M. D. & Amrhein, T. J. Approach to Imaging in Patients with Spontaneous Intracranial Hemorrhage. *Neuroimaging Clinics of North America*. 10.1016/j.nic.2018.03.003 (2018). 10.1016/j.nic.2018.03.003 30007750

[48] Kwah, L. K. & Diong, J. National Institutes of Health Stroke Scale (NIHSS). *Journal of Physiotherapy*. 10.1016/j.jphys.2013.12.012 (2014). 10.1016/j.jphys.2013.12.012 24856948

[49] Schmidt, F. A., Liotta, E. M., Prabhakaran, S., Naidech, A. M. & Maas, M. B. Assessment and comparison of the max-ICH score and ICH score by external validation. *Neurology*. 10.1212/WNL.0000000000006117 (2018). 10.1212/WNL.0000000000006117 PMC6139815 30068631

[50] Broderick, J. P., Adeoye, O. & Elm, J. Evolution of the Modified Rankin Scale and Its Use in Future Stroke Trials. *Stroke*. 10.1161/STROKEAHA.117.017866 (2017). 10.1161/STROKEAHA.117.017866 PMC5552200 28626052

