# Supplementary material for: Circulating TIMP-1 is associated with hematoma volume in patients with spontaneous intracranial hemorrhage
Source: Sci Rep. 2020 Jun 25;10:10329. doi: 10.1038/s41598-020-67250-9 (PMC7316718; doi:10.1038/s41598-020-67250-9)
Supplement: Supplementary file 1 — Supplementary information. [file 41598_2020_67250_MOESM1_ESM.docx]

**Circulating TIMP-1 is associated with hematoma volume in patients with**

**spontaneous intracranial hemorrhage**

Manuel Navarro-Oviedo, Roberto Muñoz-Arrondo, Beatriz Zandio, Juan Marta-Enguita2,

Anna Bonaterra-Pastra, Jose Antonio Rodríguez, Carmen Roncal, Jose Antonio

Páramo, Estefania Toledo, Joan Montaner, Mar Hernández-Guillamon, Josune Orbe

**Supplemental Material**

**Supplemental Figure 1.** Schematic representation of the tail-bleeding model and infusion regime

**
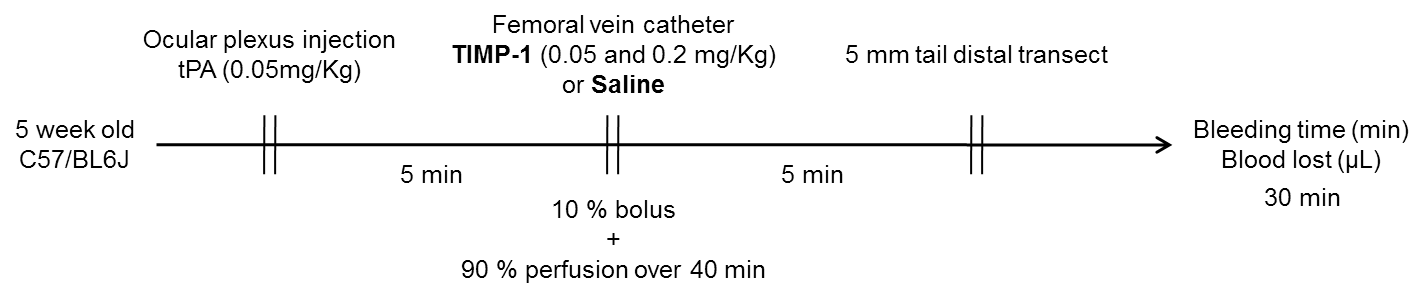
**

**Supplemental Table 1**. Associations of admission MMPs and TIMP-1 levels and cardiovascular risk factors and outcomes in the CHN cohort (n=29). Modified Rankin Scale (mRs), Hypertension (HTA), Diabetes Mellitus (DM).

| **Age** | **B (95% CI)** | **Beta** | **p value** |
| --- | --- | --- | --- |
| MMP-1 (ng/mL) | 0.175 (-0.555-0.904) | 0.101 | 0.625 |
| MMP-2 (ng/mL) | 0.036 (-0.084-0.157) | 0.124 | 0.539 |
| MMP-7 (ng/mL) | -0.103 (-0.806-0.600) | -0.061 | 0.765 |
| MMP-9 (ng/mL) | -0.030 (-0.067-0.008) | -0.310 | 0.115 |
| MMP-10 (pg/mL) | 0.011 (-0.005-0.026) | 0.277 | 0.162 |
| TIMP-1 (ng/mL) | 0.025 (-0.009-0.058) | 0.281 | 0.140 |

| **mRs 90 days** | **B (95% CI)** | **Beta** | **p value** |
| --- | --- | --- | --- |
| MMP-1 (ng/mL) | 0.073 (-0.093-0.239) | 0.191 | 0.372 |
| MMP-2 (ng/mL) | 0.009 (-0.012-0.029) | 0.179 | 0.393 |
| MMP-7 (ng/mL) | -0.060 (-0.178-0.058) | -0.220 | 0.302 |
| MMP-9 (ng/mL) | -0.001 (-0.008-0.005) | -0.095 | 0.651 |
| MMP-10 (pg/mL) | 0.000 (-0.002-0.003) | 0.061 | 0.773 |
| TIMP-1 (ng/mL) | 0.005 (-0.001-0.010) | 0.323 | 0.100 |

| **Sex (Female)** | **OR (95% CI, logistic regression)** | **p value** |
| --- | --- | --- |
| MMP-1 (ng/mL) | 1.053 (0.934-1.187) | 0.401 |
| MMP-2 (ng/mL) | 0.982 (0.960-1.004) | 0.114 |
| MMP-7 (ng/mL) | 0.924 (0.806-1.059) | 0.256 |
| MMP-9 (ng/mL) | 0.993 (0.986-1.001) | 0.086 |
| MMP-10 (pg/mL) | 0.998 (0.996-1.001) | 0.278 |
| TIMP-1 (ng/mL) | 0.997 (0.989-1.005) | 0.448 |

| **HTA (Yes)** | **OR (95% CI, logistic regression)** | **p value** |
| --- | --- | --- |
| MMP-1 (ng/mL) | 0.991 (0.883-1.113) | 0.884 |
| MMP-2 (ng/mL) | 1.011 (0.991-1.031) | 0.294 |
| MMP-7 (ng/mL) | 1.040 (0.929-1.164) | 0.494 |
| MMP-9 (ng/mL) | 0.999 (0.993-1.005) | 0.814 |
| MMP-10 (pg/mL) | 0.999 (0.996-1.001) | 0.354 |
| TIMP-1 (ng/mL) | 0.999 (0.993-1.004) | 0.698 |

| **DM (Yes)** | **OR (95% CI, logistic regression)** | **p value** |
| --- | --- | --- |
| MMP-1 (ng/mL) | 0.980 (0.848-1.133) | 0.785 |
| MMP-2 (ng/mL) | 0.984 (0.958-1.011) | 0.245 |
| MMP-7 (ng/mL) | 1.014 (0.899-1.145) | 0.816 |
| MMP-9 (ng/mL) | 0.997 (0.989-1.005) | 0.422 |
| MMP-10 (pg/mL) | 1.000 (0.997-1.003) | 0.893 |
| TIMP-1 (ng/mL) | 0.996 (0.986-1.007) | 0.482 |

| **Mortality 90 days (Yes)** | **OR (95% CI, logistic regression)** | **p value** |
| --- | --- | --- |
| MMP-1 (ng/mL) | 1.144 (0.957-1.368) | 0.140 |
| MMP-2 (ng/mL) | 1.008 (0.988-1.029) | 0.433 |
| MMP-7 (ng/mL) | 1.014 (0.905-1.137) | 0.810 |
| MMP-9 (ng/mL) | 1.000 (0.994-1.007) | 0.898 |
| MMP-10 (pg/mL) | 1.000 (0.997-1.003) | 0.970 |
| TIMP-1 (ng/mL) | 1.005 (0.997-1.014) | 0.213 |

**Supplemental Table 2**. Associations of admission MMPs and TIMP-1 levels with cardiovascular risk factors and outcomes in the VdH cohort (n=76). Modified Rankin Scale (mRs), Hypertension (HTA), Diabetes Mellitus (DM). Bold numbers indicate statistically significant p-values.

|  | **B (95% CI)** | **Beta** | **p value** |
| --- | --- | --- | --- |
| **MMP-2 (ng/mL) and age** | 0.952 (0.267 – 1.638) | 0.31 | **0.007** |
| **MMP-2 (ng/mL) and mRs 90 days** | 3.772 (0.477 – 7.067) | 0.28 | **0.025** |
| **MMP-10 (pg/mL) and age** | 11.678 (1.449 – 21.906) | 0.26 | **0.025** |
| **MMP-9 (ng/mL) and sex (female)** | -52.917 (-98.112 – 7.722) | -0.262 | **0.022** |

|  | **OR (95% CI, logistic regression)** | **p value** |
| --- | --- | --- |
| **MMP-2 (ng/mL) and HTA (yes)** | 1.032 (1.002 – 1.064) | **0.039** |
| **MMP-10 (pg/mL) and DM (yes)** | 1.001 (1.000 – 1.003) | **0.021** |
| **MMP-2 (ng/mL) and mortality 90 days (yes)** | 1.025 (1.003 – 1.048) | **0.027** |
